# Prevalence and determinants of moderate-to-severe anaemia in the third trimester of pregnancy: a multicenter cross-sectional study in Lagos, Nigeria

**DOI:** 10.1038/s41598-024-61487-4

**Published:** 2024-05-18

**Authors:** Kehinde S. Okunade, Festus O. Olowoselu, Olufemi A. Oyedeji, Yusuf A. Oshodi, Aloy O. Ugwu, Ayokunle M. Olumodeji, Adebola A. Adejimi, Muisi A. Adenekan, Temitope Ojo, Iyabo Y. Ademuyiwa, Victoria Adaramoye, Austin C. Okoro, Atinuke Olowe, Hameed Adelabu, Olukayode O. Akinmola, Salimat Yusuf-Awesu, Ayodeji A. Oluwole

**Affiliations:** 1 Department of Obstetrics & Gynaecology, College of Medicine, University of Lagos/Lagos University Teaching Hospital, PMB 12003, Lagos, Nigeria; 2 Department of Obstetrics & Gynaecology, Lagos University Teaching Hospital, Lagos, Nigeria; 3 Center for Clinical Trials, Research and Implementation Science, College of Medicine, University of Lagos, Surulere, Lagos, Nigeria; 4 Department of Haematology and Blood Transfusion, College of Medicine, University of Lagos, Lagos, Nigeria; 5 Department of Obstetrics & Gynaecology, Lagos State University Teaching Hospital, Ikeja, Lagos, Nigeria; 6 Department of Obstetrics & Gynaecology, 68 Nigerian Army Reference Hospital, Yaba, Lagos, Nigeria; 7 Department of Community Health & Primary Care, College of Medicine, University of Lagos, Surulere, Lagos, Nigeria; 8 Department of Obstetrics & Gynaecology, Lagos Island Maternity Hospital, Lagos Island, Lagos, Nigeria; 9 Department of Obstetrics & Gynaecology, Federal Medical Center, Ebute-Meta, Lagos, Nigeria; 10 Department of Nursing Science, College of Medicine, University of Lagos, Surulere, Lagos, Nigeria; 11 Department of Chemical Pathology, Lagos University Teaching Hospital, Surulere, Lagos, Nigeria

**Keywords:** BMI, Lagos, Moderate-to-severe anaemia, Predictors, Predict-PPH, Health care, Risk factors

## Abstract

The high burden of anaemia during pregnancy underscores the urgent need to gain a comprehensive understanding of the factors contributing to its widespread occurrence. Our study assessed the prevalence and the trends of moderate-to-severe anaemia (MSA) in late pregnancy (28 to 36 weeks) and then investigated the key determinants driving this prevalence among women in Lagos, Nigeria. We conducted a secondary data analysis involving 1216 women enrolled in the *Predict-PPH* study between January and March 2023. We employed a multivariate binary logistic regression model with a backward stepwise selection approach to identify significant predictors of MSA. The study revealed a 14.5% prevalence of MSA during pregnancy. Independent predictors of MSA included having given birth to two or more children (adjusted odds ratio = 1.46, 95% confidence interval: 1.03–2.07), having a maternal body mass index (BMI) of 28 kg/m^2^ or higher (adjusted odds ratio = 1.84, 95% confidence interval: 1.29–2.61), having less than tertiary education (adjusted odds ratio = 1.51, 95% confidence interval: 1.08–2.11), and being unemployed (adjusted odds ratio = 1.97, 95% confidence interval: 1.19–3.26). It is crucial for pregnant women, particularly those with higher parities and elevated BMI, to be monitored regularly for anaemia and its consequences during their antenatal care. Additionally, addressing the link between low education, unemployment, and anaemia necessitates comprehensive strategies that empower women in terms of education and economic status to enhance the overall well-being of individuals and communities, ultimately reducing the prevalence of anaemia and associated health issues in pregnancy.

## Introduction

Anaemia is an indicator of both poor nutrition and poor health. Its occurrence in pregnancy may result in women’s impaired health, and quality of life, and consequential impairment in newborn and child’s development and learning^1^. Anaemia is the most common medical disorder of pregnancy^2^ and a major contributor to maternal and perinatal morbidity and mortality worldwide^3^. It is a decrease in the concentration of circulating haemoglobin (Hb) in the peripheral blood, below the level that is considered sufficient for one's age, gender, and geographical location^4^. According to the World Health Organization (WHO), anaemia is typically characterized by Hb levels lower than 11.0 g/dL at any stage of pregnancy and less than 10.0 g/dL postpartum^5^.

Based on WHO estimates, globally, 41.8% of pregnant women experience anaemia, with a notably higher prevalence on the African continent, where approximately 56% of pregnant women are identified as anaemic^6^. This is even more staggering within the context of pregnancy due to the physiological changes in the second trimester that lead to an elevation in plasma volume, accompanied by a relatively smaller increase in red cell mass, resulting in hemodilution—manifesting as ‘physiological anaemia’^7^. According to the 2018 Nigerian Demographic and Health Survey (NDHS), approximately 58% of pregnant women in Nigeria grapple with this condition^8^.

In sub-Saharan Africa (SSA), an Hb level below 10.0 g/dL is frequently employed as a criterion for diagnosing anaemia during pregnancy^4^. This cut-off has been justified based on the work of Lawson et al.^9^, which suggested that serious harm to the mother and fetus hardly occurs above this cut-off value, which is regarded as moderate to severe levels according to the WHO classification^10^. Globally, 23% of maternal mortality is attributed to moderate-to-severe anaemia (MSA) during pregnancy, particularly occurring at 28 weeks and beyond^11^. The magnitude and burden of MSA vary globally. It is estimated that about 37% (32 million) of pregnant women aged 15–49 years were affected by anaemia, with roughly half of these cases attributed to moderate to severe levels^12^. This condition is a significant public health concern, especially in developing countries, and can lead to increased morbidity and mortality, particularly in pregnant women and their newborns^13^.

The high burden of anaemia in pregnancy, therefore, emphasizes the pressing need to comprehensively understand the determinants behind the ubiquity of MSA during pregnancy. Furthermore, the third trimester is a crucial period in pregnancy because the demand for iron increases as the fetus undergoes rapid growth and development in utero^14^. Therefore, in this study, we delved into the multifaceted landscape of MSA in pregnancy by determining the prevalence and the trends of MSA, and then explored the intricate web of determinants that drive this prevalence among women in the third trimester of pregnancy (28 to 36 weeks) in Lagos, Nigeria. By shedding light on these critical factors, we aim to contribute to a deeper understanding of this pressing global health issue and pave the way for effective strategies for the prevention and management of MSA in a resource-limited setting such as Nigeria.

## Participants and methods

### Study design and settings

This was a descriptive cross-sectional study of pregnant women enrolled at baseline in the recently conducted *“Predict-PPH”* study^15^. “*Predict-PPH*” was a prospective cohort study of consecutively consenting healthy pregnant women aged 15–49 years and at 28–36 weeks’ gestation enrolled at the antenatal clinics of five hospitals in Lagos, Nigeria from January to June 2023^15^. These were the Lagos University Teaching Hospital (LUTH) in Idi-Araba, Federal Medical Center Ebute Meta (FMC-Eb), 68 Nigerian Army Reference Hospital (68-NARHY) in Yaba, Lagos Island Maternity Hospital (LIMH) in Lagos Island, and Lagos State University Teaching Hospital (LASUTH) in Ikeja. These hospitals are the foremost public health institutions in Lagos State, Southwest Nigeria, with a population of over 20 million inhabitants, and they act mainly as referral centres for other government-owned and private hospitals in Lagos and its surrounding States. They all have established obstetrics and gynaecology departments with comprehensive maternity units that account for a cumulative annual delivery of 15,700^15^. Over 90% of payments for health services in these hospitals are usually made directly out-of-pocket without insurance while approximately 10% of the remaining clients pay through some form of social health insurance including the Lagos State Ilera Eko Scheme (LSIES) and the National Health Insurance Scheme (NHIS)^16^.

### Eligibility criteria

We included and analyzed the data of n = 1216 women with complete datasets collected at baseline enrollment from the primary study^15^. Excluded at enrolment were women with a major medical condition such as sickle cell anaemia, significant renal and hepatic impairment, coagulation disorders and antepartum fetal demise. Further exclusion in this current study were women whose haemoglobin (Hb) concentration was not recorded at enrollment.

### Extracted variables of interest

Variables extracted for analyses in the dataset included site and type of enrolment facility, participant’s age in years, gestational age at enrolment in weeks, number of previous childbirths, body mass index (BMI) in kg/m^2^, marital, educational and employment status, mode of conception, type of pregnancy, antepartum bleeding in index pregnancy, ultrasound diagnosis of uterine fibroids and Hb concentration at enrolment in g/dL determined using HemoCue® B-Hemoglobin system. Body mass index (BMI; calculated as maternal weight [using the actual pre-gestational or first-trimester measurement] in kilograms divided by the square of height in meters)^17^. As recommended by the WHO^10^, participants were categorized based on their Hb concentration as non-anaemic (≥ 11 g/dL), mild anaemia (10 to < 11 g/dL), moderate anaemia (7 to < 10 g/dL) and severe anaemia (< 7 g/dL).

### Operational definitions of study outcomes

We assessed two study outcomes: the prevalence of moderate-to-severe anaemia (MSA), defined as the proportion of study participants with Hb concentration less than 10 g/dL^10^, and determinants of MSA, which are the baseline variables from the *Predict-PPH* study^15^ that are significantly associated with MSA.

### Sample size calculation

Using Fisher's formula^18^, we estimated that a sample size of n = 308 would be required to determine the prevalence of MSA based on a type I error rate of 5% at a 95% confidence level of 1.96 and a derived proportion of 27.6%^4^. In addition, to identify predictors of MSA, we estimated the sample size using the maximum modelling principle proposed by Peduzzi et al.^19^. This required that a minimum of 10 events (women with MSA in pregnancy) would occur for each incorporated prediction variable given that the prevalence of MSA is 27.6%^4^ and that model building included 12 potential predictor variables from the dataset. A sample size of n = 458 women was required to identify independent predictors of MSA in pregnancy assuming a non-response or data recording error rate of 5%. We, therefore, included all the n = 1216 women who had their complete datasets at baseline in the primary study^15^ in the statistical analyses. In the original study^15^, there was no predetermined allocation of participants to the study sites. The number (and proportion) of participants from each study site was based on the volume of pregnant women seen and who consented to enrolment at each site during the study period.

### Statistical analysis

We tested continuous variables for normality using the Kolmogorov–Smirnov test with Lilliefors’ significance correction and descriptive statistics were then computed for the participants’ clinical and obstetric characteristics. Categorical variables were expressed as frequencies and percentages, whereas continuous variables were displayed as mean (± standard deviation) for normally distributed data or median (interquartile range) for skewed distributions. Bivariate analyses of variables that are potential predictors of MSA in pregnancy were performed using Pearson’s Chi-square test. A multivariate binary logistic regression model was then developed using a stepwise selection approach to identify significant predictors of MSA in pregnancy. Variables that were associated with MSA in pregnancy (*P* < 0.10) in the bivariate analyses were included in the pool of variables for the backward stepwise regression model. An Akaike’s Information Criterion was generated constantly and the last model step with the smallest AIC was selected as the best-fit model. Associations in the final model were regarded as significant if *P* < 0.05. Data analyses were performed with IBM SPSS Statistics for Windows, Version 28.0 (IBM Corporation, Armonk, NY, USA).

### Ethical considerations

Approval for the primary study^15^ was granted by the Health Research Ethics Committees of the Lagos University Teaching Hospital (ADM/DSCST/HREC/APP/5443), Lagos State University Teaching Hospital (LREC/06/10/2000) and Institute of Tropical Medicine, Antwerp (IRB/RR/AC/002). The research was conducted ethically according to the World Medical Association Declaration of Helsinki. Before enrollment, all study participants provided written informed consent, and a rigorous commitment to maintaining the privacy and confidentiality of participant information was upheld throughout and after the conduct of the study.

## Results

A total of n = 1216 women who had their complete datasets out of the 1222 women enrolled at baseline in the primary study^15^ were included in the data analyses. Out of these, n = 176 (14.5%) had MSA. The characteristics of the enrolled cohorts are presented in Table 1.

**Table 1:** Characteristics of participants (n = 1216).

Characteristics	Number (%)
Mean age (± SD) in years	30.7 ± 5.3
Mean gestation age at enrolment (± SD) in weeks	31.8 ± 2.5
Mean BMI (± SD) in kg/m^2^	27.9 ± 5.9
Enrolment site	
LUTH	148 (12.2)
LASUTH	160 (13.2)
LIMH	519 (42.7)
FMC-Eb	185 (16.2)
68-NARHY	204 (16.8)
Level of enrolment facility	
Secondary	723 (59.5)
Tertiary	493 (40.5)
Previous childbirths	
≥ 2	364 (29.9)
< 2	852 (70.1)
Marital status	
Unmarried	15 (1.2)
Married	1201 (98.8)
Educational status	
At least tertiary education	832 (68.4)
Less than tertiary education	384 (31.6)
Employment status	
Unemployed	96 (7.9)
Employed	1120 (92.1)
Mode of conception	
Assisted	18 (1.5)
Spontaneous	1198 (98.5)
Type of pregnancy	
Multiple	26 (2.1)
Singleton	1190 (97.9)
Antepartum bleeding in the index pregnancy	
Yes	106 (8.7)
No	1110 (91.3)
Presence of uterine fibroids	
Yes	150 (12.3)
No	1066 (87.7)
Anaemia status	
No (≥ 11 g/dL)	648 (53.5)
Mild (10–< 11 g/dL)	387 (32.0)
Moderate (7–< 10 g/dL)	175 (14.4)
Severe (< 7 g/dL)	1 (0.1)

BMI, body mass index; FMC-Eb, Federal Medical Center Ebute-Meta; IQR, interquartile range; LASUTH, Lagos State University Teaching Hospital; LIMH, Lagos Island Maternity Hospital; LUTH, Lagos University Teaching Hospital; 68-NARHY, 68 Nigerian Army Reference Hospital Yaba; SD, standard deviation.

**Figure 1:**
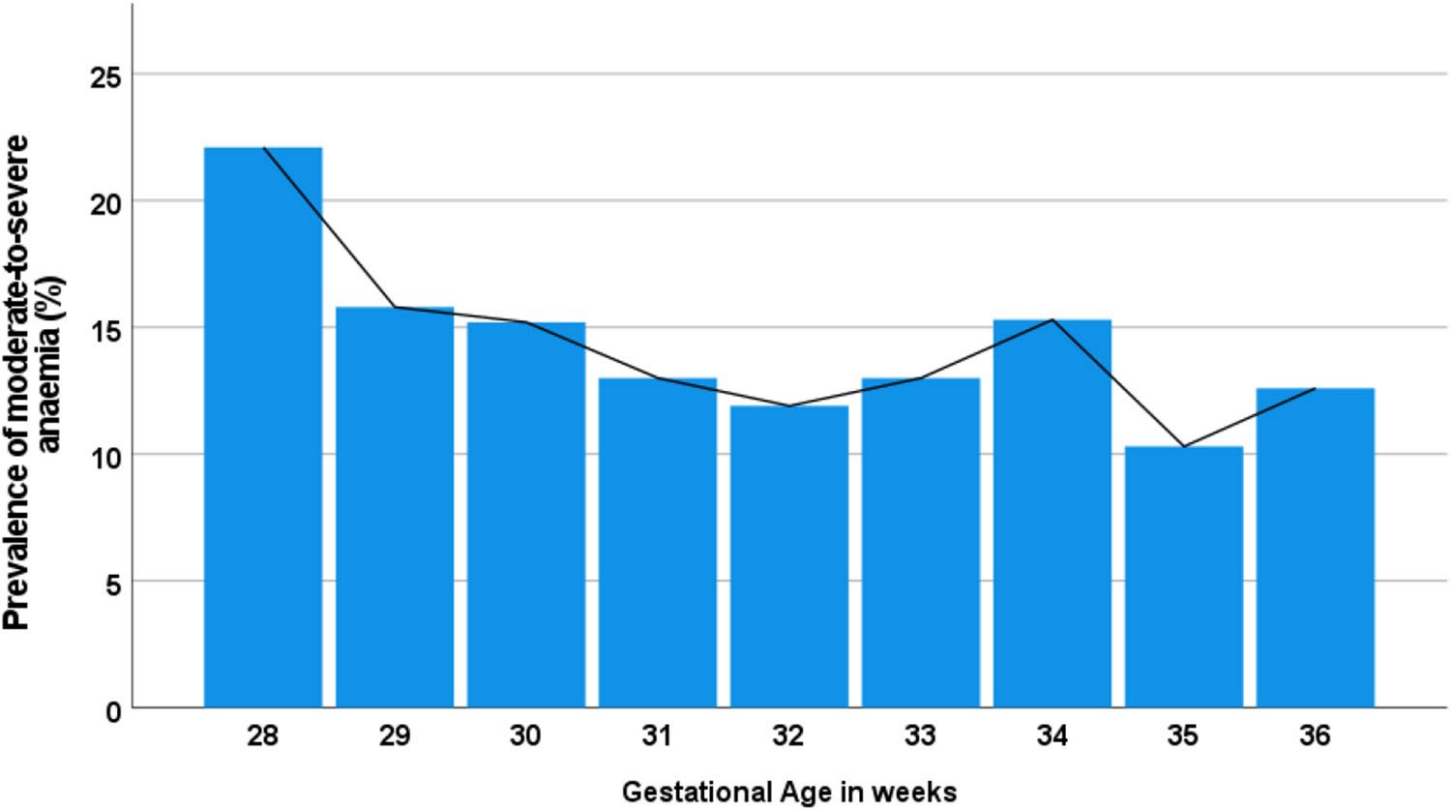
Trend in the prevalence of moderate-to-severe anaemia from 28 to 36 weeks gestational age.

Figure 1 showed a progressive reduction in the prevalence of MSA from 22.5% at 28 weeks to 12.5% at 36 weeks’ gestation.

In the bivariable analyses, factors that were significantly associated with the MSA were: enrolment gestational age, number of previous childbirths, increased BMI, and educational and employment status. In the final multivariable analysis, the following predictor variables were independently associated with MSA: previous childbirths ≥ 2 (adjusted odds ratio = 1.46, 95% confidence interval: 1.03–2.07), maternal BMI ≥ 28 kg/m^2^ (adjusted odds ratio = 1.84, 95% confidence interval: 1.29–2.61), having less than tertiary education (adjusted odds ratio = 1.51, 95% confidence interval: 1.08–2.11), and being unemployed (adjusted odds ratio = 1.97, 95% confidence interval: 1.19–3.26) (Table 2).

**Table 2:** Bivariable and multivariable analyses of potential predictors of moderate-to-severe anaemia (n = 1216).

Predictor	Moderate-to-severe anaemia		Bivariate *p*-value	OR (95% CI)	Multivariable *p*-value
	Yes (%)	No (%)			
Number of participants	176 (14.5)	1040 (85.5)	NA	NA	NA
Level of enrolment facility					
Secondary	67 (13.6)	426 (86.4)	0.470	NA	NA
Tertiary	109 (15.1)	614 (84.9)			
Enrolment gestational age					
≥ 32 weeks	97 (16.4)	495 (83.6)	0.065	1.31 (0.94–1.81)	0.108
< 32 weeks	79 (12.7)	545 (87.3)		1.00	Reference
Participants age					
≥ 31 years	95 (14.7)	552 (85.3)	0.825	NA	NA
< 31 years	81 (14.2)	488 (85.8)			
Previous childbirths					
≥ 2	62 (17.0)	302 (83.0)	0.097	1.46 (1.03–2.07)	0.033
< 2	114 (13.4)	738 (86.6)		1.00	Reference
BMI					
≥ 28 kg/m^2^	122 (17.5)	576 (82.5)	< 0.001	1.84 (1.29–2.61)	< 0.001
< 28 kg/m^2^	54 (10.4)	464 (89.6)		1.00	Reference
Marital status					
Married	172 (14.3)	1029 (85.7)	0.177	NA	NA
Unmarried	4 (26.7)	11 (73.3)			
Educational status					
Less than tertiary education	73 (19.0)	311 (81.0)	0.002	1.51 (1.08–2.11)	0.016
At least tertiary education	103 (12.4)	729 (87.6)		1.00	Reference
Employment status					
Unemployed	24 (25.0)	72 (75.0)	0.002	1.97 (1.19–3.26)	0.008
Employed	152 (13.6)	968 (86.4)		1.00	Reference
Mode of conception					
Assisted	2 (11.1)	16 (88.9)	0.683	NA	NA
Spontaneous	174 (14.5)	1024 (85.5)			
Type of pregnancy					
Multiple	3 (11.5)	23 (88.5)	0.667	NA	NA
Singleton	173 (14.5)	1017 (85.5)			
Antepartum bleeding					
Yes	16 (15.1)	90 (84.9)	0.849	NA	NA
No	160 (14.4)	950 (85.6)			
Uterine fibroids					
Yes	24 (16.0)	126 (84.0)	0.570	NA	NA
No	152 (14.3)	914 (85.3)			

BMI, body mass index; CI, confidence interval; NA, not applicable; OR, adjusted odds ratio.

## Discussion

In this descriptive cross-sectional study of healthy pregnant women enrolled at baseline in the *Predict-PPH* study, one in seven of the women were reported as having moderate-to-severe anaemia (MSA) during pregnancy. The prevalence of MSA reduced progressively between 28- and 36-weeks’ gestation. Independent predictors of MSA included having given birth to two or more children, having a high pre- or early-pregnancy body mass index (BMI), having less than tertiary education, and being unemployed.

The prevalence of 14.5% for MSA reported in our study is similar to the 15.3% found by Uche-Nwachi et al.^20^ in a study of 2287 pregnant women attending 40 public healthcare centers in Trinidad and Tobago between January 2000 and December 2005 and the global prevalence of 16.5% reported by the World Health Organization (WHO)^12^ based on antenatal Hb assessments done at the end of the second trimester. However, this is notably lower than the 25% seen among pregnant women in Kakamega county of Kenya^21^ and the 27.6% reported in another Lagos in 2014^4^. This discrepancy may suggest a gradual reduction in pregnancy-related anaemia due to increased awareness and compliance with antenatal iron supplementation, particularly among women enrolled in our clinics. This variation could also be attributed to the study’s location in an urban area of Lagos metropolis with predominantly higher socioeconomic status participants^22^, contributing to the remarkably low anaemia prevalence.

Furthermore, we observed a trend towards decreasing MSA prevalence from 22.5% at 28 weeks’ gestation to 12.5% at 36 weeks. This contrasts with the findings from a previous study by Okunade et al. in Lagos^4^ where anaemia was less common in the 2nd trimester. Similarly, another Lagos study by Anorlu et al.^23^ and an East African study conducted by Chrispinus et al. in Kakamega county in Kenya^21^ reported significantly higher anaemia rates of anaemia in the second and third trimesters, particularly among pregnant women who had recently registered for antenatal care. Previous research has validated that anaemia tends to be exacerbated by the physiological haemodilution characteristic of pregnancy, with a more noticeable impact in the last two trimesters^24^. However, the reduced prevalence with advancing gestational age in our study may suggest better compliance with antenatal care, supplements, and nutrition among pregnant women as their pregnancies approach full term.

The association we identified between higher parity and MSA in pregnancy aligns with the findings by Al-Farsi et al. in Oman^25^ and a study by Gedefaw et al. in Ethiopia^26^, which demonstrated that women with high-parity pregnancies had a higher risk of anaemia, often showing a dose–response relationship across multiple categories of parity. This association may be due to the shorter intervals between pregnancies commonly observed in our setting, limiting the body's ability to replenish nutrient stores, including iron, depleted from previous pregnancies. Additionally, it may be explained by the increased demand for nutrients to support a growing fetus with each successive pregnancy.

Several studies have reported that lower BMI in pre- or early pregnancy is associated with anaemia in pregnancy^27,28^ due to the possible link of low BMI with poor nutritional intake, involving the consumption of diverse micronutrients crucial for hematopoiesis^29^ and chronic illness, such as tuberculosis or parasitic infections, ultimately resulting in anaemia^26^. However, we reported an independent association between maternal BMI ≥ 28 kg/m^2^ and anaemia. This link may be attributed to factors such as hypoferremia due to dilution, insufficient dietary iron intake, increased iron requirements, diminished iron absorption^30^, and elevated inflammation resulting from increased hepcidin levels. This, in turn, diminishes iron absorption from the intestines in obese individuals^31^.

The high prevalence of anaemia has been associated with low socio‐economic status^21,32^. Therefore, as highlighted in our study, two major indices of social status including having less than tertiary education and being unemployed were independently predictive of MSA. This underscores the interconnected relationship between low education, unemployment, poverty, and poor nutritional outcomes, including anaemia, especially in resource-limited settings such as Nigeria^33^. Women living in poverty often face financial limitations, limiting their access to safe, sufficient, and nutritious food, as well as quality and affordable antenatal care^32,33^. Therefore, our study reinforces the belief and findings from previous research that nutritional deficiency particularly that of iron is the leading cause of anaemia in pregnancy^34^, particularly in resource-limited countries^35^, where it is closely linked to poverty and malnutrition^36^.

The findings of this study have significant implications for clinical practice and policy. The study underscores the importance of routine screening for anaemia during antenatal visits, especially for pregnant women with identified risk factors. Clinicians should also be vigilant in assessing these risk factors and should consider individualized management strategies for at-risk pregnant women including dietary interventions, iron supplementation, and referral to specialized care if necessary. In addition, policymakers should allocate resources to ensure access to adequate antenatal care services, including routine screening for anaemia, particularly among vulnerable populations. This may involve strengthening healthcare infrastructure, providing training for healthcare professionals, and ensuring the availability of affordable and accessible iron supplementation. The study has a substantial sample size which provided robust evidence to support the findings and the multi-center settings of the study ensured that the findings are representative of the broader population of pregnant women who attend antenatal care in the Lagos metropolis.

Despite its numerous strengths, the study has some limitations. The study findings can only be generalized primarily to the urban secondary and tertiary health institutional settings in Lagos, as the participating pregnant women were enrolled with the exclusion of those attending primary health centres as well as those living in the slums and suburban areas of Lagos. Secondly, the lack of random allocation in participants’ recruitment from the study sites could increase the risk of selection bias with certain study sites disproportionately enrolling participants with specific characteristics or risk factors which could thus compromise the generalizability of the study findings to the broader population. Furthermore, the cross-sectional nature of the study prevents us from making causal inferences regarding the observed relationships between the identified risk factors and MSA. We, therefore, recommend that future longitudinal studies should be designed to employ a more comprehensive sampling strategy that includes women from a wider range of clinical and community settings, as well as those residing in slums and suburban areas to allow a trajectory tracking of anaemia during pregnancy and assess the temporal relationship between potential risk factors and the development of MSA. These studies should also conduct more in-depth analyses of potential risk factors for MSA, including socio-demographic factors, dietary habits, and underlying health conditions as a way of informing targeted interventions to address individual women-specific risk profiles, especially before conception.

## Conclusions

We reported that one in seven women had MSA in late pregnancy and identified independent predictors of MSA as having had two or more previous childbirths, a high maternal BMI, having less than a tertiary education, and being unemployed. We also recorded a progressive reduction in the prevalence of MSA between 28- and 36-weeks’ gestation. It is, therefore, crucial for pregnant women, particularly those with higher parities and elevated BMI, to be regularly monitored for anaemia during their antenatal care. Additionally, addressing the link between low education, unemployment, and anaemia necessitates comprehensive strategies that empower women in terms of education and economic status to enhance the overall well-being of individuals and communities, ultimately reducing the prevalence of anaemia and associated health issues in pregnancy.

## Data Availability

The datasets used and/or analyzed during the current study are available from the corresponding author (KSO) upon reasonable request.
